# Effects of Functionalized and Raw Multi-Walled Carbon Nanotubes on Soil Bacterial Community Composition

**DOI:** 10.1371/journal.pone.0123042

**Published:** 2015-03-31

**Authors:** Dorsaf Kerfahi, Binu M. Tripathi, Dharmesh Singh, Hyoki Kim, Sujin Lee, Junghoon Lee, Jonathan M. Adams

**Affiliations:** 1 Department of Biological Sciences, Seoul National University, Seoul, Republic of Korea; 2 School of Chemical and Biological Engineering, Interdisciplinary Program of Bioengineering, Seoul National University, Seoul, Republic of Korea; 3 Celemics, Inc. 612 Avison Biomedical Research Center, Yonsei Medical Center, Seoul, Republic of Korea; 4 School of Mechanical and Aerospace Engineering, Interdisciplinary Program of Bioengineering, Seoul National University, Seoul, Republic of Korea; National University of Mongolia, MONGOLIA

## Abstract

Carbon nanotubes (CNTs) are widely used in industry, but their environmental impacts on soil microbial communities are poorly known. In this paper, we compare the effect of both raw and acid treated or functionalized (fCNTs) multi-walled carbon nanotubes (MWCNTs) on soil bacterial communities, applying different concentrations of MWCNTs (0 µg/g, 50 µg/g, 500 µg/g and 5000 µg/g) to a soil microcosm system. Soil DNA was extracted at 0, 2 and 8 weeks and the V3 region of the 16S rRNA gene was PCR-amplified and sequenced using paired-end Illumina bar-coded sequencing. The results show that bacterial diversity was not affected by either type of MWCNT. However, overall soil bacterial community composition, as illustrated by NMDS, was affected only by fMWCNT at high concentrations. This effect, detectable at 2 weeks, remained equally strong by 8 weeks. In the case of fMWCNTs, overall changes in relative abundance of the dominant phyla were also found. The stronger effect of fMWCNTs could be explained by their intrinsically acidic nature, as the soil pH was lower at higher concentrations of fMWCNTs. Overall, this study suggests that fMWCNTs may at least temporarily alter microbial community composition on the timescale of at least weeks to months. It appears, by contrast, that raw MWCNTs do not affect soil microbial community composition.

## Introduction

Carbon nanotubes (CNTs) are widely used in novel industrial materials because of their particular chemical and physical characteristics [1–3]. They are currently used for—or under development in—electron emission devices, energy storage devices, drug delivery mechanisms, and a range of other engineering applications [4–9]. Carbon nanotubes (CNTs) are single atom layers of hexagonal carbon rolled up into hollow cylinders. They are classified as single-walled carbon nanotubes (SWCNTs) and multi-walled carbon nanotubes (MWCNTs). SWCNTs are single-layered graphitic cylinders with a diameter ranging from 0.4 to 2 nm and MWCNTs which are composed by 2 to 30 concentric cylinders with outer diameters ranging between 2 and 100 nm [10]. Previous studies have demonstrated that SWCNTs are more toxic to human and animal cells, whereas MWCNTs exhibit a milder toxicity [11,12], indicating that the diameter of CNTs was a key factor governing their toxic activity [11]. Furthermore, short MWCNTs exhibited significant toxicity when they were uncapped, debundled, and dispersed in solution [13]. Liu et al. [14] showed that SWCNTs dispersed individually in solution were more toxic to bacteria than aggregated SWCNTs, because individually dispersed SWCNTs can behave as numerous moving “nano darts” and can constantly attack bacterial cells, causing degradation then death of bacterial cell [15].

Global carbon nanotube production is increasing by around 25% per year and by 2015 is expected to reach 9300 tons with a production value of $1.3 billion [16]. Nevertheless, there are no strict rules regulating CNTs production, usage and release. Consequently, substantial quantities of CNTs could be released into the environment with the potential to affect the environment and human health [17]. There is still limited knowledge on the actual and potential production volume as well as the release of CNTs in the environment [18]. Moreover, the currently predicted low average released concentrations will slowly increase due to increased CNTs production and use [19,20]. The release of CNTs to the environment may occur during the production phase or the usage and disposal phases. Direct or indirect exposure pathways to CNTs have rarely been studied, and the risks to human health and the general environment are still poorly understood [21]. To assess the environmental risks presented by CNTs, it is important to understand their fate after release (their mobility, reactivity and persistence in environmental compartments), and their impact on living organisms [22].

Despite their evident advantages in practical applications, the potential toxicity of CNTs is a major concern because of their potential impact in the environment [23]. CNTs’ toxicity has been studied in both *in vivo* and *in vitro*, and has been related to various factors such as CNTs’ length, type of functionalization, concentration, duration of exposure, method of exposure, concentration of the solubilizing agent, and the surfactant used. So far, while many studies suggest that CNTs do not show toxicity, certain others suggest that CNTs are harmful to human health and the environment. These inconsistencies might be due to differences in experimental protocol [24].

Up to the present, few studies have investigated the impact of CNTs on living organisms in the environment [2,25,26]. Most ecotoxicological studies that have dealt with their effects on bacteria have been conducted under culture conditions [11,27]. Only few studies have investigated the influence of these nanoparticles on microbial community *in situ* [28,29]. For example, Kang et al. [30], demonstrated that the exposure of *Escherichia coli* to highly purified CNT aggregates can lead to cell death. In other studies, it has been shown that CNTs dispersed using a range of different surfactants can have antimicrobial properties when incubated with bacteria [14,31]. It has been demonstrated that carbon nanotubes can also lower soil enzyme activities and microbial biomass when applied to soil [2,32,33].

Soil is likely to be one of the main ultimate recipients of nanomaterial contamination in the environment, more so than water and air [25,26,34]. Soil microbial communities play a vital role in soil ecosystem activities such as nutrient cycling, and are known to be susceptible to alteration by heavy metals and a range of other chemical agents [14,35,36]. Therefore, it is important to study the impact of CNTs on these living soil systems [2,12,17]. If it were to turn out that CNTs strongly alter the composition or functioning of the soil ecosystem, and that the effects persist over the long term, precautionary measures for their manufacture, usage and disposal could be necessary.

Previous studies on CNTs in the soil system have been carried out by Chung et al. [2] who demonstrate that short-term exposure (20 days) to multi-walled CNTs can lower most enzymatic activities and overall microbial biomass in soils, at exposures of around 5000 μg of MWCNTs per gram of soil. The same group [32] has observed similar effect of CNTs on soil enzyme activities and microbial biomass at the concentration 300–1000 μg/g but using single-walled CNTs (SWCNTs). Another short-term study indicates that SWCNTs may alter the structure of activated sludge microbial communities [37]. Furthermore, many of the previous studies used untreated CNTs, which are known to be hydrophobic, mixing poorly with soils. Only a subset of studies has used the far more easily miscible acid–treated CNTs (functionalized or fCNTs), which are also commonly used in industry [32], and which seem more likely to interact with the soil ecosystem.

Here we set out to understand the effect of both treated (fMWCNTs) and untreated MWCNTs on soil bacterial communities at a range of taxonomic levels. We consider shifts in both relative abundance and diversity. The relative abundance of certain groups might be important because particular taxa are consistently associated with ecological functions in the soil, especially at the finer taxonomic level. Diversity may be important because it is widely thought that ecosystem resilience is affected by taxonomic diversity [38–40].

Furthermore, we continued our experiment for 8 weeks; most other studies ran for less than 4 weeks [2,32]. Given that soil bacteria are generally thought to be slow growing with a high proportion of dormant cells much of the time, a longer duration of study seems desirable, being more likely to show relevant shifts in ecology. Shrestha et al. [17] evaluated the impact of MWCNTs on soil microbial community structure and functioning in soil over 90 days of exposure and they found no effect on soil respiration, enzymatic activities and microbial community composition. However, at the highest (MWCNTs) concentration (10,000 mg/kg), shifts in microbial community composition and abundance of some bacterial genera were observed.

Essentially our hypotheses were as follows:

1. That fMWCNTs will have a greater effect on soil bacteria than untreated MWCNTs, due to their greater ability to mix with soil water and interact directly with bacterial cells. This higher concentration of MWCNTs will also have stronger effects.
2. That given the relatively slow and long-term nature of soil processes, including bacterial community shifts, the 8-week timeframe will show a different result from the much shorter 2–3 week timeframe used in most previous studies. The effect may intensify, as more cells die or are unable to reproduce under the effect of the MWCNTs.

In the present study, we use paired-end Illumina bar-coded sequencing of hypervariable V3 region of 16S rRNA gene to investigate the impact of multi-walled carbon nanotubes on soil bacteria.

## Materials and Methods

### Soil sampling

Soil samples were collected in June 2013 from an overgrown flowerbed on Seoul National University campus, which is located in the Gwanak Mountain area, south of Seoul. This sampling site was selected because it represents a typical and widespread type of soil (slightly acidic sandy loam) in South Korea. The upper 10 cm of soil was sieved and thoroughly mixed in a sterile container. The soil was divided into pots containing 200 g soil each. Soil pH was measured using a soil pH meter (Hanna Instruments HI 99121N Direct Soil pH Meter). Soil pH was around 6.1 and it contained 10.4% clay, 18.4% silt and 71.2% sand. Soil texture and organic matter content were measured at National Instrumentation Center for Environmental Management (NICEM, South Korea) following the standard protocol of SSSA (Soil Science Society of America).

### Preparation of MWCNTs

Pure commercial MWCNTs were purchased from Hanwha Nanotech, Republic of Korea. Two forms of CNTs were used in the present experiment: raw and functionalized forms of MWCNTs. The powder MWCNTs were not treated, but used in the form received from the purchaser because this is a form that microorganisms might encounter if there is an accidental release from a manufacturing facility. The functionalized MWCNTs are more commonly used during fabrication processes of commercial products, so would more likely be released at those sites [32].

The MWCNTs were functionalized (fMWCNTs), following the protocol described by Saito et al. [41], by attaching carboxyl groups (-COOH) to their surfaces using acidic solutions [42]. A mixture of H_2_SO_4_:HNO_3_ = 3:1 (v:v) were added to the raw MWCNTs at room temperature [43]. The mixture was bath sonicated for 24h, followed by vacuum filtration through 0.22 μm Millipore Teflon membrane (JGWP04700). Then, the membrane was thoroughly washed using deionized (DI) water, and was immersed in DI water according to established protocols [44]. The MWCNTs were then dried overnight in the oven at 60°C.

### Characterization of MWCNTs

MWCNTs were characterized using Energy-Filtering transmission electron microscopy (EF-TEM: LIBRA 120, Carl Zeiss, Germany) and field-emission scanning electron microscopy (FE-SEM: S-4800, Hitachi, Japan). These techniques were effective in characterizing the internal structure (diameter and wall number) of MWCNTs. Raman spectra were taken to determine the diameter distribution using LabRam Aramis (Horiba Jobin-Yvon, France).

The metal components in the MWCNTs are less than 5% in weight. They have been analyzed by the manufacturer (Hanwha Nanotech, Republic of Korea) and are aluminum, iron, and molybdenum.

### Soil incubation

The soil was divided into plastic self-draining pots containing 200 g soil each, to give 3 replicates to be exposed to each concentration of raw MWCNTs or fMWCNTs. The concentrations of MWCNTs applied to soil were 0 (DI water only), 50, 500, and 5000 μg/g soil. The soils were then well mixed to ensure homogeneity before incubation in a BOD incubator at 25°C for 8 weeks. The pots were not covered to allow free gas exchange to the soil microbial community. The positions of replicate pots of different treatments were randomized and randomly interchanged each week. Soil moisture was adjusted to 60% water holding capacity. Soil moisture content was maintained by weighing the pots twice a week and adjusting to initial weight by regular addition of DI water. Samples of 3 g of soil were collected from each pot, at different time points (0, 2, and 8 weeks), to be used for DNA extraction. At T = 0 weeks, we took samples almost immediately (1 hour later) after adding MWCNTs to soil.

### DNA extraction and sequencing

The soil DNA was extracted from 0.3 g of the mixed 3 g sample of soil, using the Power Soil DNA extraction kit (MO BIO Laboratories, Carlsbad, CA, USA) following the protocol described by the manufacturer. DNA isolated from each sample was amplified using primers 338F (5 = -XXXXXXXXGTACTCCTACGGGAGGCAGCAG-3 =) and 533R (5 = TTACCGCGGCTGCTGGCAC-3 =), targeting the V3 hypervariable regions of the bacterial 16S rRNA gene (the X sequence denotes a barcode sequence) [45]. The Polymerase chain reactions (PCR) were carried out under the following thermal profile: denaturation at 94°C for 2 min, followed by 25 cycles of amplification at 94°C for 30 s, 57°C for 30 s and 72°C for 30 s, followed by a final extension of 72°C for 5 min. PCR products were analyzed by electrophoresis in 1% agarose gels and were purified using Wizard SV Gel and PCR Clean-up System (Promega, USA). The paired-end sequencing was performed at Kim lab incorporation (Yonsei University, Seoul), using a paired 150-bp HiSeq 2000 sequencing system (Illumina) according to the manufacturer’s instructions. Library preparation, sequencing and initial quality filtering were performed as described previously [46].

### Quantitative PCR analysis

Relative abundance of bacterial subunit rRNA gene copies was quantified using quantitative PCR (qPCR). Standard curves were created using a 6-fold serial dilution (10^-2^ to 10^-7^) of a plasmid containing a full-length copy of the *Escherichia coli* 16S rRNA gene, to estimate bacterial relative abundance. qPCR assays were conducted in 48-well plates. Each 10 μl reaction contained 5 μl of reaction mixture (2X Real-Time PCR Smart mix), 0.5 μl of forward and reverse primers (Eub 338 and Eub 518), and DNA-free water. PCR conditions were 2 min at 50°C, and 15 min at 95°C, followed by 40 cycles of 95°C for 60 s, 53°C for 30 s and 72°C for 45 s. Melting curve analyses was performed to confirm that the amplified products were of the appropriate size. Each plate included triplicate reactions per DNA sample.

### Data analysis

The sequenced data were processed using the mothur platform [47]. Illumina sequencing data was pair-assembled using pandaseq [48] with an assembly quality score of 0.9, which is the most stringent option to reduce errors. Next, the sequences were aligned against the EzTaxon-aligned reference [49]. Sequences were denoised using the ‘*pre*.*cluster*’ command in mothur, which applies a pseudo-single linkage algorithm with the goal of removing sequences that are likely due to pyrosequencing errors [50]. Putative chimeric sequences were detected and removed via the Chimera Uchime algorithm contained within mothur [51]. The taxonomic classification was performed using mothur’s version of the RDP Bayesian classifier, using EzTaxon-e database for each sequence at 80% Naïve Bayesian bootstrap cutoff with 1000 iterations. The sequences used in this study have been deposited in the NCBI Sequence Read Archive under accession number SRP043977.

### Statistical analysis

To perform the statistical analysis, all samples were standardized by random subsampling to 4,073 sequences per sample, using the sub.sample command (http://www.mothur.org/wiki/Sub.sample) in mothur. To assess the relationship between soil bacteria richness/diversity and MWCNTs concentration, as well as with time incubation, the richness of OTUs and other diversity indices were calculated using the mothur platform [47].

We calculated weighted UniFrac which measures sequence difference between samples based on phylogenetic information to analyze the bacterial community similarity. We used a non-metric multidimensional scaling plot (NMDS) using the weighted UniFrac distance in Primer 6 to visualize the clustering of bacterial community composition over time. We then performed an analysis of similarity (ANOSIM) with pairwise weighted UniFrac distance as the response variable and MWCNTs concentration, time incubation and form as factors. We performed multiple regression analysis in R software package 2.15.2 using linear model (LM) for normal data, and a generalized linear model (GLM) for non-normal data to evaluate the effects of raw and functionalized MWCNTs concentrations, incubation time, and their interactions on bacterial richness and diversity, as well as on the relative abundance of dominant bacterial phyla. There were four different concentration treatments of raw and functionalized MWCNTs i.e. 0, 50, 500, and 5000 μg/g soil, and each treatment had three replicates. Aliquots of soil from each treatment were collected at different time points (0, 2, and 8 weeks). To test whether bacterial abundance (qPCR) was correlated with fMWCNTs and raw MWCNTs across different sampling time, we performed regression analysis using linear functions in SigmaPlot. We used analysis of variance (ANOVA) to test the effect of fMWCNTs and raw MWCNTs on organic matter content of the soils.

## Results

A total of 2,568,331 quality bacterial sequences were obtained from the 63 samples, with an average of 40,767 sequences per soil sample and with coverage ranging from 4,760 to 219,775 reads per sample (S1 Table).

### MWCNTs characterization

**Fig 1:**
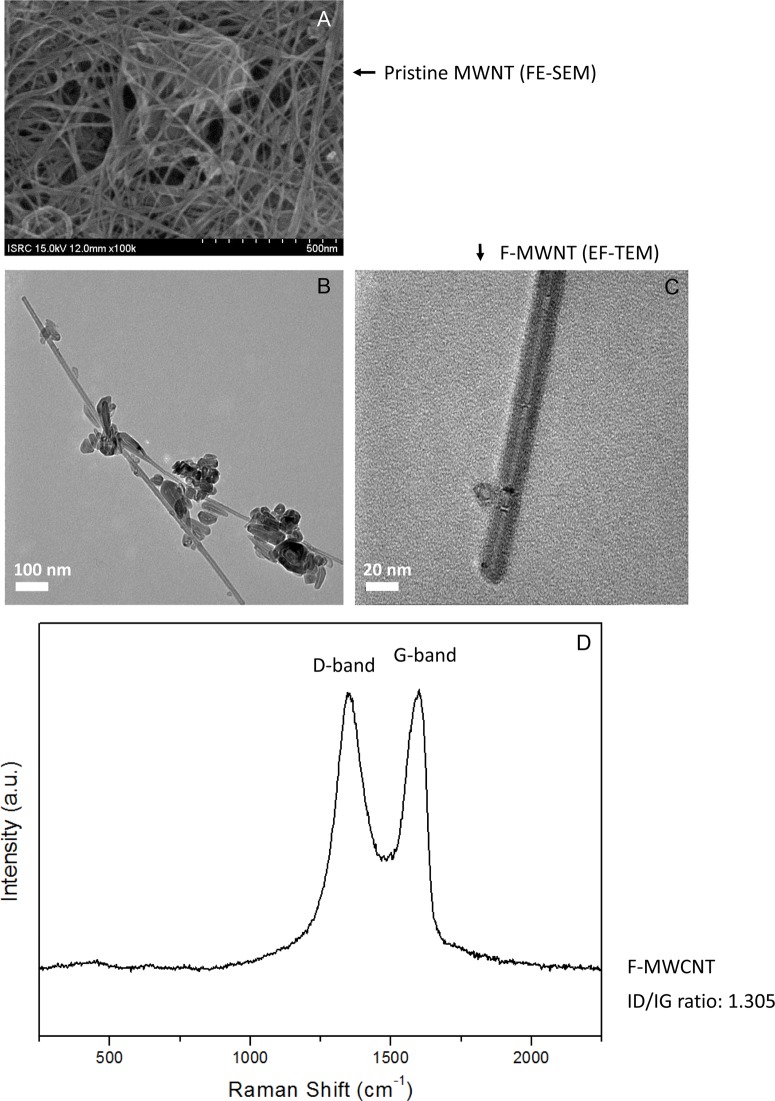
Characterization of multi-walled carbon nanotubes (MWCNTs) used in the study. (A) Field-emission scanning electron microscope (FE-SEM) image of the MWCNTs. (B,C) Energy-filtering transmission electron microscope (EF-TEM) images of the MWCNTs. (D) Raman spectrum of the MWCNTs.

The characterization of MWCNTs based on FE-SEM (Fig. 1A) and EF-TEM (Fig. 1B, 1C) images showed that the average diameter was around 13.4 nm and the number of walls was 11 in average. The size of CNTs is an important factor in toxicological studies [11,52]. In fact, the interactions between carbon nanotubes and living cells decreased with the size increase [11].

Raman spectrum showed that the D-band/G-band ratio was approximately 1.305 and this result showed that defects have been generated from pristine MWCNT which D-band/G-band ratio is 1.087 (Fig. 1D).

### Effect of MWCNTs on bacterial community diversity

The effects of raw and functionalized MWCNTs concentrations, incubation time, and their interactions on bacterial richness and diversity were evaluated using multiple regression analyses. The results showed that the concentration of both fMWCNTs and raw MWCNTs did not show any correlation with OTUs richness and Chao index (All P > 0.05). In contrast, time was significantly correlated with OTUs richness and diversity for the two MWCNTs forms (All P < 0.05). Considering time and MWCNTs concentration together, there was an important correlation for OTUs richness and diversity indices (All P < 0.05), but only for functionalized carbon nanotubes (fMWCNTs) (Table 1).

**Table 1:** Multiple regression between richness (OTUs) and diversity indices with CNTs concentrations and incubation time for both acid treated (fMWCNTs) and raw MWCNTs.

fMWCNTs		OTUs (R^2^ = 0.54***)	Shannon (R^2^ = 0.53***)	Simpson (R^2^ = 0.58***)	Chao (R^2^ = 0.52***)
	Intercept	1.745e^+03^ ***	6.5520***	7.585e^-02^ ***	6086.9***
	Time	-4.144e^+01^ ***	-5.808e^-02^ **	3.330e^-03^ **	-229.7***
	CNTs conc	-1.599e^-02^	-2.563e^-05^	7.721e^-07^	-0.1159
	Time*CNTs conc	-8.115e^-03^	-1.394e^-05^ *	1.460e^-06^ **	-0.0195
Raw MWCNTs		OTUs (R^2^ = 0.83***)	Shannon (R^2^ = 0.86***)	Simpson (R^2^ = 0.76***)	Chao (R^2^ = 0.75***)
	Intercept	1.815e^+03^ ***	6.6820***	-5.4760***	6.390e^+03^ ***
	Time	-6.114e^+01^ ***	-8.951e^-02^ ***	1.283e^-01^ ***	-3.197e^+02^ ***
	CNTs conc	-8.303e^-04^	1.250e^-05^	-4.735e^-05^	-1.071e^-01^
	Time*CNTs conc	-1.711e^-03^	-3.182e^-06^	1.027e^-05^	-6.929e^-03^

Significance level is shown at

***P ≤ 0.001,

** P ≤ 0.01, and

* P ≤ 0.05.

### Effect of MWCNTs on bacterial community composition

**Fig 2:**
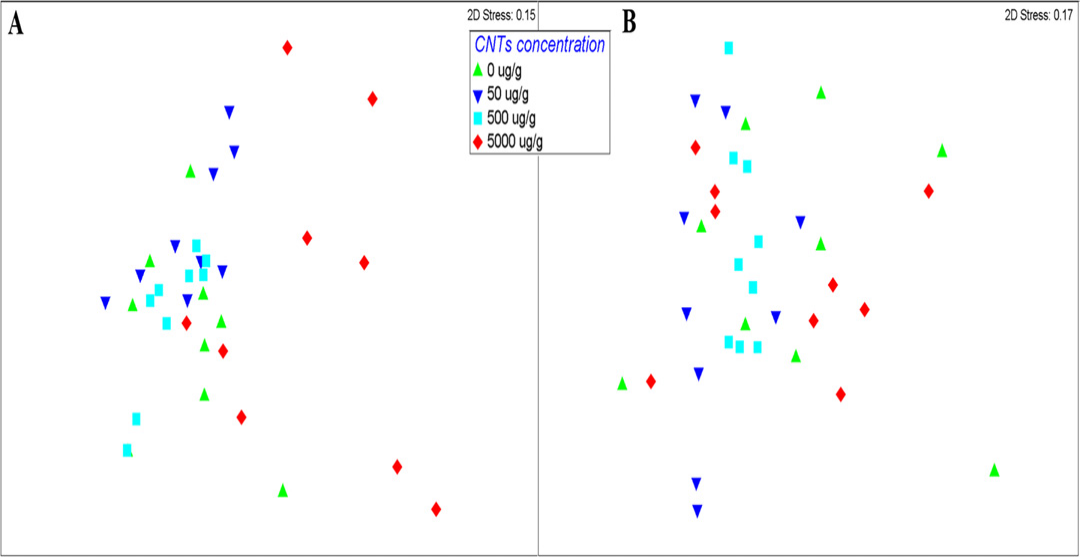
NMDS of Bray-Curtis Index of bacterial community composition in relation to (A) fMWCNTs and (B) raw MWCNTs concentrations applied to soil among the different treatments.

OTU community composition did however differ between different concentrations of fMWCNTs, (fMWCNTs, R = 0.24, P = 0.001) but not with different concentrations of raw MWCNTs for Bray-Curtis dissimilarities. NMDS plots showed a clustering of soil samples according to MWCNT concentrations; nevertheless samples from soil with highest concentration of fMWCNTs showed a greater dissimilarity in their bacterial community composition compared to soil treated with raw MWCNTs (Fig. 2).

**Fig 3:**
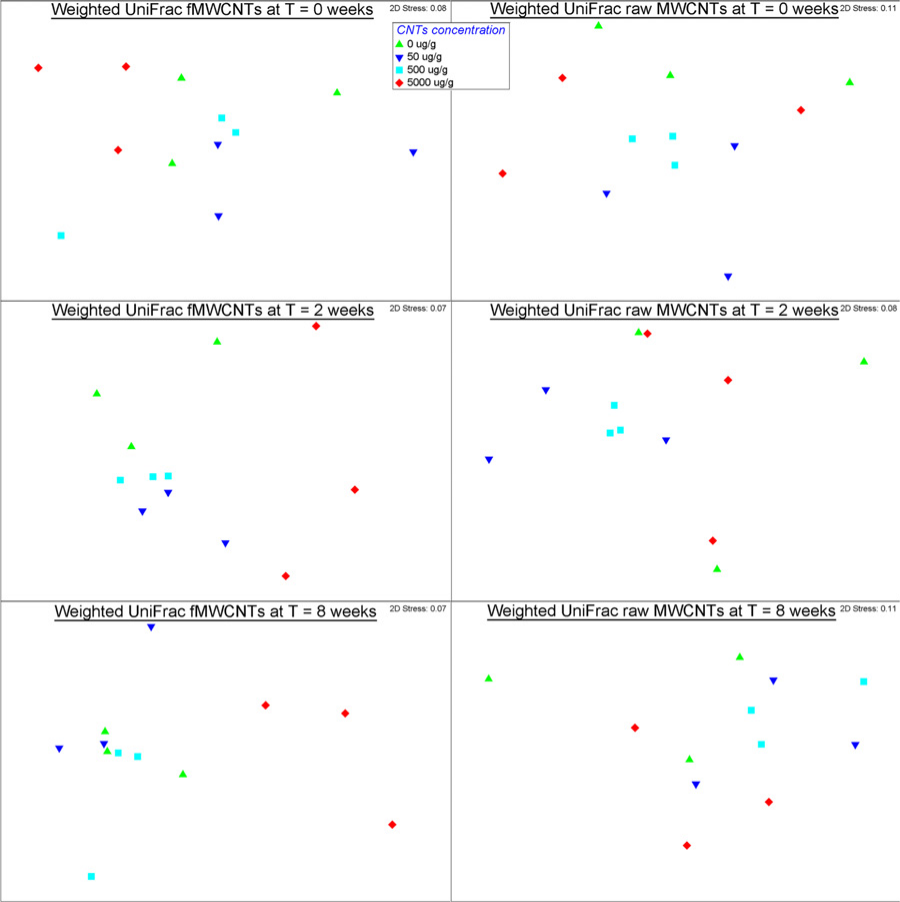
NMDS of weighted UniFrac indices of bacterial community composition in relation to MWCNTs concentrations applied to soil over time (at T = 0 weeks, 2 weeks, and 8 weeks).

Considering the variation in the bacterial community over time, the NMDS plot showed a clustering of the soils sampled at different sampling times according to different MWCNT concentrations used in this experiment for fMWCNTs, but not for raw MWCNTs (Fig. 3). An ANOSIM test confirmed this result as indicated by the test results (Table 2).

**Table 2:** ANOSIM results for weighted UniFrac dissimilarity over time.

Form of MWCNTs	0 weeks		2 weeks		8 weeks	
	R value	P value	R value	P value	R value	P value
fMWCNTs	0.21	0.015	0.66	0.006	0.72	0.003
Raw MWCNTs	0.10	0.07	-0.015	0.63	0.27	0.3

### Effect of MWCNTs on bacterial community abundance

Overall, the most abundant bacterial phyla were *Proteobacteria* with 29% of the sequences, followed by *Acidobacteria* (20%), *Actinobacteria* (15%), *Chloroflexi* (9%), and *Bacteroidetes* (7%); around 4% of the sequences were unclassified.

**Fig 4:**
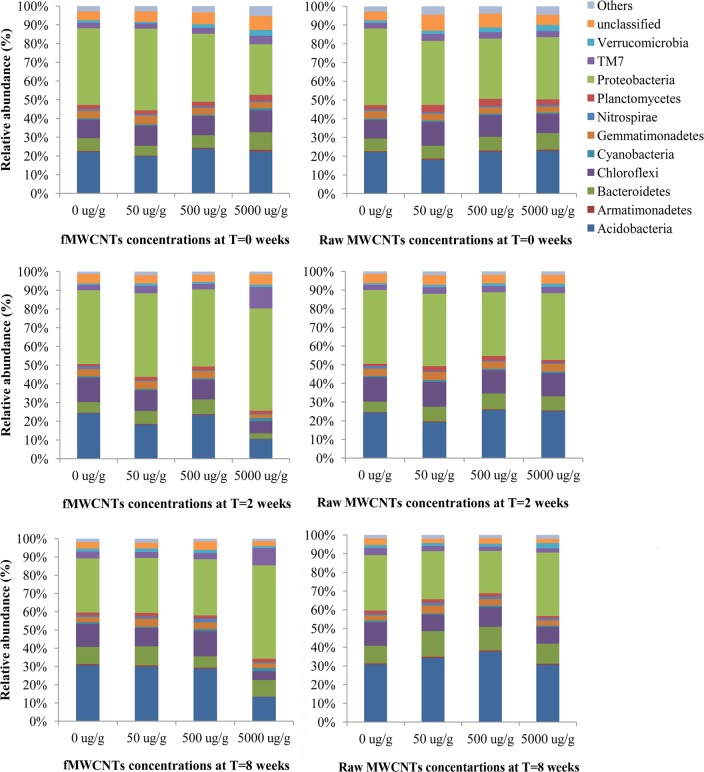
Bacterial composition of the most abundant phyla among different concentrations of fMWCNTs and raw MWCNTs over time (at T = 0 weeks, 2 weeks and 8 weeks).

Of the most abundant phyla, we found significant differences in relative abundance between different concentrations of fMWCNTs except for *Proteobacteria*, *Actinobacteria*, *Chloroflexi*, and *Bacteriodetes* (Fig. 4). The multiple regression analyses showed that time and fMWCNTs together have an effect on soil bacteria (Table 3). However, the samples treated with raw MWCNTs showed less correlation with both time and concentration for some phyla (Table 3 and Fig. 4). Consequently, the fMWCNTs showed a more highly significant change in the relative abundance of the dominant detected phyla.

**Table 3:** Multiple regression between the relative abundance of the dominant bacterial phyla and with the concentration and incubation time of fMWCNTs and raw MWCNTs.

fMWCNTs		*Acidobacteria*(R^2^ = 0.51***)	*Proteobacteria*(R^2^ = 0.46***)	*Actinobacteria*(R^2^ = 0.20*)	*Bacteroidetes*(R^2^ = 0.06)	*Chloroflexi*(R^2^ = 0.55***)
	Intercept	17.2028***	34.8423***	17.2211***	5.0180***	8.7270***
	Time	1.1080**	-1.1033***	-0.6582	3.304e^-01^ *	2.502e^-01^ *
	CNTs conc	-0.0005	-0.0019***	0.0009	7.732e^-05^	-1.746e^-04^
	Time*CNTs conc	-0.0003**	0.0001	0.0001	-5.961e^-05^	-1.706e-^04^ ***
Raw MWCNTs		*Acidobacteria*(R^2^ = 0.58***)	*Proteobacteria*(R^2^ = 0.31**)	*Actinobacteria*(R^2^ = 0.40***)	*Bacteroidetes*(R^2^ = 0.50***)	*Chloroflexi*(R^2^ = 0.01)
	Intercept	4.0410***	3.093e^+01^ ***	1.733e^+01^ ***	5.1920***	9.9320***
	Time	1.802e^-01^ ***	-9.371e-01***	-9.190e^-01^ ***	6.627e^-01^ ***	-2.775e^-02^
	CNTs conc	7.403e^-05^	-5.010e^-04^	-5.049e^-04^	3.126e^-04^	-7.906e^-05^
	Time*CNTs conc	-1.699e^-05^	2.102e^-04^ *	1.245e^-04^	-7.143e^-05^	-3.151e^-05^

Significance level is shown at

***P ≤ 0.001,

** P ≤ 0.01, and

* P ≤ 0.05.

**Fig 5:**
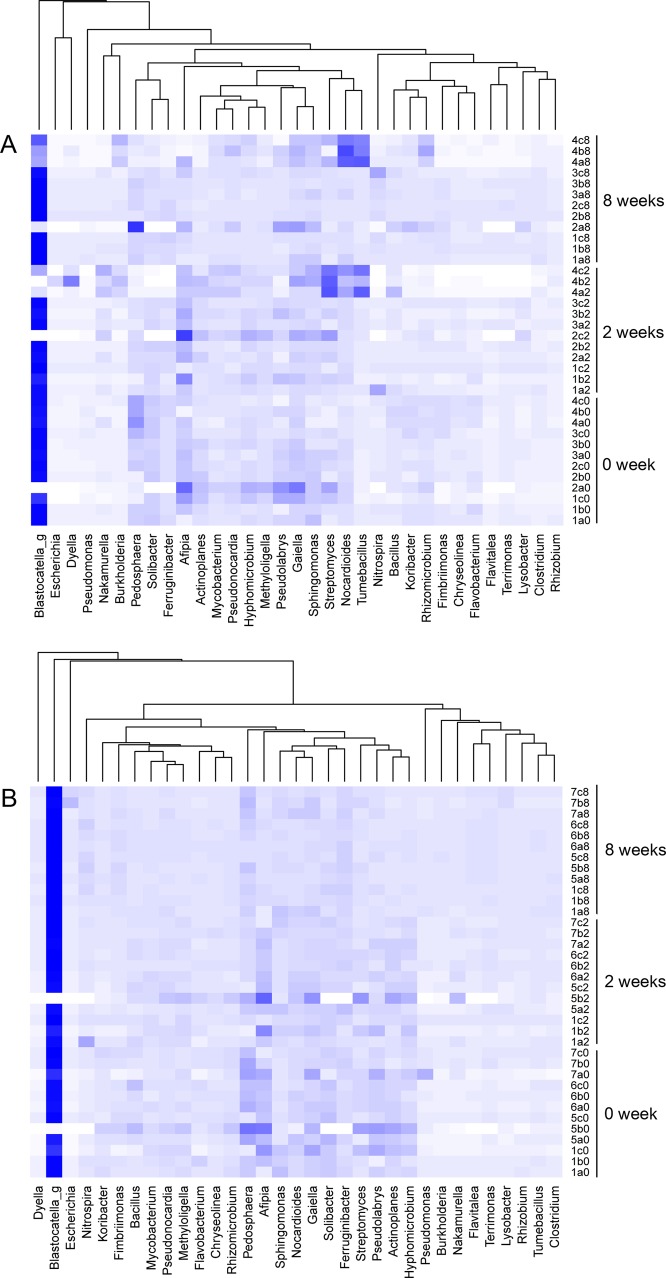
The heat map showing the relative abundances of the most abundant genera at different MWCNTs concentrations applied to soil over time (at T = 0 weeks, 2 weeks, and 8 weeks). The numbers written on Y-axis indicate the concentration of MWCNTs (1: 0 μg/g, 2: 50 μg/g, 3: 500 μg/g, 4: 5000 μg/g of fMWCNTs and 5: 50 μg/g, 6: 500 μg/g, 7: 5000 μg/g of raw MWCNTs), and the letters indicate the replicates.

Changes in the relative abundance of the predominant bacterial genera in response to exposure to fMWCNTs and raw MWCNTs are illustrated in Fig. 5 using a heat map. The most abundant OTU across the various soil replicates/treatments was classified under the genus *Blastocatella* (Acidobacteria) represented by 5.6% of the total reads. This genus significantly decreased in abundance in the highest treatments with fMWCNTs at 2 weeks, but later in the experiment its abundance increased by the final time of sampling at 8 weeks. However, raw MWCNTs did not have any detectable effect on OTU relative abundances. Overall, even fMWCNTs did not have profound effects on the soil bacterial community, even though effects were detectable. Shifts in bacterial abundance were observed only for fMWCNTs with the greatest changes observed at highest concentrations (5000 μg/g).

**Fig 6:**
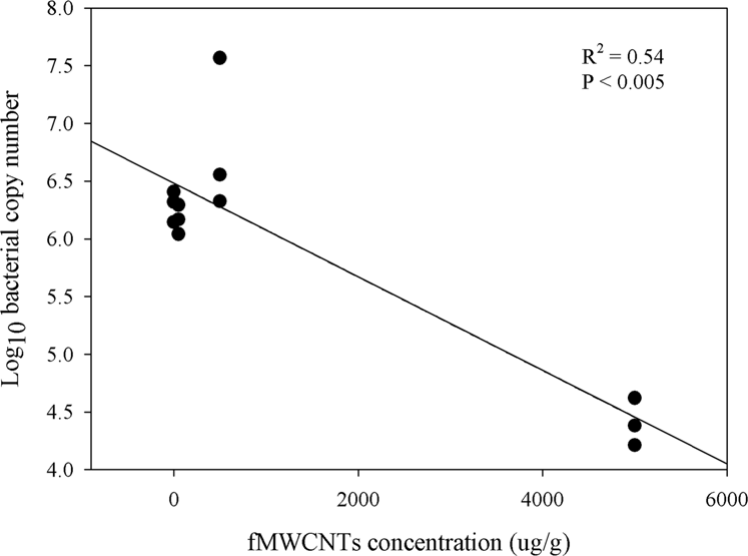
Relationship between fMWCNTs concentrations and bacterial copy numbers at T = 0 weeks.

For all the qPCR assays, there was a linear relationship between the log of plasmid DNA copy number and the calculated threshold cycle value across the different concentration range (R^2^ > 94 in all cases). The bacterial abundance, as determined using qPCR, did not show any correlation with fMWCNTs or raw MWCNTs across different concentrations, except with fMWCNTs at zero time where P < 0.005 and R^2^ = 0.54 (Fig. 6).

### Effect of MWCNTs on soil organic matter

The ANOVA test results showed that the content of organic matter measured in the soil among different treatments with MWCNTs were highly significant (F_3,8_ = 58.08, P < 0.001). High concentrations of both forms of MWCNTs increased the organic matter content of the soil.

## Discussion

### Effect of MWCNTs on soil bacterial community

In our study, we had predicted that MWCNTs would significantly alter both the soil bacterial community and its diversity. Yet during the two month-long experiment, we found no differences in diversity as a result of exposure to either raw or fMWCNTs. Nevertheless, fMWCNTs showed an effect on bacterial community composition. Raw MWCNTs did not show any effect on community composition. Changes in the structure and abundance of the soil bacterial community in response to MWCNT exposure have been observed in previous studies [17,37]. Our results showed that soils treated with fMWCNTs exhibited shifts in bacterial community composition for different MWCNT concentrations at both of the sampling times (2 weeks and 8 weeks).

The relative abundance of the most common bacterial phyla was affected by the presence of fMWCNTs and showed differing responses to exposure to fMWCNTs. The abundance of *Proteobacteria*, and *TM7* was comparatively higher in the highest fMWCNTs treatment, while there was a decrease in *Chloroflexi* at the highest concentration of fMWCNTs. *Acidobacteria*, *Bacteriodetes* and *Gemmatimonadetes* showed an initial decrease (2 weeks) at the highest concentration of fMWCNTs then increased over time (8 weeks), whereas *Actinobacteria* increased at highest concentration of fMWCNTs, then later decreased. These findings suggest that the exposure of soil to fMWCNTs could in fact have some impact on carbon cycling by altering the microbial community [12]. For example, *Actinobacteria* (which undergo significant shifts in abundance) are important in the biogeochemical cycle of carbon in soils [53,54]. In particular, they play a major role in the degradation of cellulolytic and hemicellulolytic compounds in soils [55,56]. *Acidobacteria* are ubiquitous and among the most abundant bacterial phyla in soil [57]: their relative abundance is generally negatively correlated with soil carbon availability [58]. The *Chloroflexi* are commonly found in soils, also playing an important role in the biogeochemical cycle of carbon and the CO_2_ dynamics in soils [55,56,59]. Such changes in bacterial abundance may be explained in terms of a shift of microbial community towards bacterial species that are more tolerant of the effects of fMWCNTs and the decline of less tolerant species [60–62].

Despite the evident effects of exposure to fMWCNTs, overall it appears from our experiment that the soil bacterial community is quite resilient to the environmental perturbation caused by high concentrations of fMWCNTs. The community largely recovers from exposure to fMWCNTs by 8 weeks. It also appears that the soil bacterial community is resistant to perturbation from raw MWCNTs, with almost no observed effects on bacterial community. The observed lack of response to raw MWCNTs generally matches previous findings for untreated nanotubes and other carbon-based new materials [63,64]. For instance, Khodakovskaya et al. [65] observed no impact on soil bacterial diversity of MWCNTs added by watering into soil at concentrations up to 200 μg/ml. Other studies on the impact of a carbon-based nanomaterial, C_60_ fullerene by Chung et al. [2] and Tong et al. [66] demonstrated no effect of toxicity on soil bacterial diversity even at 1000 mg/kg concentration.

However, some studies have shown toxin-like effects of raw MWCNTs on bacteria. Rodrigues et al. [12] found that raw MWCNTs can negatively affect soil bacterial diversity. In fact, they observed a major effect of single-walled carbon nanotubes on the soil bacterial community after only 3 days of exposure, and then bacterial diversity recovered after 14 days’ exposure. If this is the case, our observed lack of an effect from raw MWCNTs after 2 weeks may be due to the system having already recovered from an initial perturbation. MWCNTs are chemically extremely inert, especially in relation to biological processes [42], and this could be one of the possible reasons that we did not find any effect of MWCNTs on soil bacterial communities.

### Why do fMWCNTs cause a shift in the soil bacterial community?

fMWCNTs are acidic in nature: pure fMWCNTs, after thorough washing, have a pH around 3 due to carboxyl groups that cover their surface. Our measurements of soil pH showed that pH was around 4 just after adding fMWCNTs to soil at 0 weeks for the highest fMWCNTs concentration, two units lower than the control without nanotubes, around 4.8 at 2 weeks, and around 5.5 at 8 weeks. There is abundant evidence that pH is crucial to bacterial community structure [67–69]; in fact, pH seems the strongest factor of all in structuring soil bacterial communities on a global scale [70,71]. Thus, it is no surprise that fMWCNTs—with associated lowering of soil pH—caused significant changes in soil bacterial communities. One might hypothesize that fMWCNTs effects would decrease or even disappear on a time scale of months as their acidity becomes neutralized. Longer-term studies are warranted to confirm whether this is indeed the case. Aside from following the overall bacterial community, it will also be important to examine effects on biogeochemical processes, such as carbon and nitrogen cycling, in fMWCNT-contaminated soils.

## Conclusion

The overall picture is of rather weak—but still detectable—effects from fMWCNTs on soil bacterial community structure, combined with the lack of any observable effects from raw MWCNTs. This gives a generally reassuring picture in terms of the effects of MWCNTs on the soil environment. Even at the high concentrations used here, fMWCNTs apparently do not have profound effects on soil bacterial communities. The observed effects of fMWCNTs would, however, warrant further experimental investigations for any changes in soil nutrient cycling processes, using functional metagenomics or observations of fluxes.

## Supporting Information

S1 Table Relative abundances of bacterial phyla classified against the Ribosomal Database Project (RDP) training dataset number 9 across all 63 soil replicates of different treatments with raw and acid treated MWCNTs. (XLSX)
